# HEALTH INFORMATION–SEEKING BEHAVIORS, CONFIDENCE, AND CHALLENGES AMONG FAMILY CAREGIVERS OF PERSONS WITH DEMENTIA

**DOI:** 10.1093/geroni/igad104.2524

**Published:** 2023-12-21

**Authors:** Jane Chung, Rebecca Davidsson

**Affiliations:** Virginia Commonwealth University, Richmond, Virginia, United States; University of New Orleans, New Orleans, Louisiana, United States

## Abstract

Family caregivers play a significant role in providing care for their loved ones with dementia, including communication with healthcare providers (HCP), treatment decision-making, and care coordination. These tasks require family caregivers’ ability to seek, manage, and interpret health information they have found or received during and outside clinical encounters. This study examined which sources family caregivers use for seeking dementia care information, how trustworthy and reliable they find these sources, and information seeking confidence and challenges. A survey designed to understand family caregivers’ information seeking was completed by 36 participants who provided at least 10 hours of dementia care weekly (mean age 63.4; 83% female). About 80% reported using the Internet to look for dementia information, and less than 20% used a smartphone app to exchange their loved one’s medical information with HCP through a protected portal. While family caregivers entrusted HCP, pharmacists, and dementia-related organizations as sources of dementia information, they were skeptical of radio and TV as sources of credible information. Other information sources were identified, including Alzheimer’s Association, support groups, books/documentaries, and research journals. Approximately 50% reported concerns about the quality of dementia-related information; 45% experienced difficulties accessing the information they needed; 44% sensed frustration while searching for dementia-related information; and 40% had difficulty understanding the information found. There should be efforts to develop strategies that help dementia caregivers better identify credible information sources to increase confidence and reduce the stress and burden of acquiring reliable information necessary to care for their loved ones.

